# Community-based bilingual doulas for migrant women in labour and birth – findings from a Swedish register-based cohort study

**DOI:** 10.1186/s12884-020-03412-x

**Published:** 2020-11-23

**Authors:** Ulrika Byrskog, Rhonda Small, Erica Schytt

**Affiliations:** 1 School of Education, Health and Social Sciences, Dalarna University, Falun, Sweden; 2 Judith Lumley Centre, La Trobe University, 3086 Melbourne, Victoria Australia; 3 Division of Reproductive Health, Department of Women’s and Children’s Health, Karolinska Institutet, Tomtebodavägen 18a, 171 77 Stockholm, Sweden; 4 Centre for Clinical Research Dalarna, Uppsala University, Nissers väg 3, 791 82 Falun, Sweden; 5 Faculty of Health and Social Sciences, Western Norway University of Applied Sciences, Møllendalsveien 6, Postboks 7030, 5020 Bergen, Norway

**Keywords:** Community-based bilingual doula support, register study, labour and birth outcomes, migrant

## Abstract

**Background:**

Community-based bilingual doula (CBD) services have been established to respond to migrant women’s needs and reduce barriers to high quality maternity care. The aim of this study was to compare birth outcomes for migrant women who received CBD support in labour with birth outcomes for (1) migrant women who experienced usual care without CBD support, and (2) Swedish-born women giving birth during the same time period and at the same hospitals.

**Methods:**

Register study based on data retrieved from a local CBD register in Gothenburg, the Swedish Medical Birth Register and Statistics Sweden. Birth outcomes for migrant women with CBD support were compared with those of migrant women without CBD support and with Swedish-born women. Associations were investigated using multivariable logistic regression, reported as odds ratios (aORs) with 95% confidence intervals (CI), adjusted for birth year, maternal age, marital status, hypertension, diabetes, BMI, disposable income and education.

**Results:**

Migrant women with CBD support (n = 880) were more likely to have risk factors for adverse pregnancy outcomes than migrant women not receiving CBD support (n = 16,789) and the Swedish-born women (n = 129,706). In migrant women, CBD support was associated with less use of pain relief in nulliparous women (epidural aOR 0.64, CI 0.50–0.81; bath aOR 0.64, CI 0.42–0.98), and in parous women with increased odds of induction of labour (aOR 1.38, CI 1.08–1.76) and longer hospital stay after birth (aOR 1.19, CI 1.03–1.37). CBD support was not associated with non-instrumental births, perineal injury or low Apgar score. Compared with Swedish-born women, migrant women with CBD used less pain relief (nulliparous women: epidural aOR 0.50, CI 0.39–0.64; nitrous oxide aOR 0.71, CI 0.54–0.92; bath aOR 0.55, CI 0.36–0.85; parous women: nitrous oxide aOR 0.68, CI 0.54–0.84) and nulliparous women with CBD support had increased odds of emergency caesarean section (aOR 1.43, CI 1.05–1.94) and longer hospital stay after birth (aOR 1.31, CI 1.04–1.64).

**Conclusions:**

CBD support appears to have potential to reduce analgesia use in migrant women with vulnerability to adverse outcomes. Further studies of effects of CBD support on mode of birth and other obstetric outcomes and women’s experiences and well-being are needed.

## Background

The introduction of community-based bilingual doulas (CBDs) is one initiative that aims to respond to migrant women’s needs and to reduce barriers to high quality maternity care [1, 2]. Migrant women’s increased risk of adverse pregnancy outcomes and some obstetric interventions such as caesarean section, is well-known [3–6]. Migrant women have also rated maternity care less positively than non-migrant women and may experience communication and language barriers, lack of familiarity with how care is provided, and discrimination and negative staff attitudes. Experiences of being left alone in labour, and feeling fearful, unsafe and unsupported have also been reported by migrant women [1].

CBDs are lay women from migrant communities, fluent in both the destination country language and the mother tongue of the pregnant migrant woman, trained to provide continuous empowering and woman-focused support that complements the role of midwives. The CBD’s language and cultural understanding facilitates communication between the woman-partner-staff during labour and birth, even though she does not replace an accredited interpreter, and helps women and their partners in navigating an unfamiliar maternity care system [7–11]. To date, results from randomised controlled trials of CBD during childbirth for migrant women have not yet been reported. Observational studies suggest that CBD support increases migrant women’s probability of a normal labour and birth [8, 9], and increased breastfeeding initiation [12]. A positive experience of care has been reported [10, 13, 14], also after a cesarean [15], and no harms have been documented [12, 13, 16]. In addition, continuous physical and emotional support in labour from a person assigned only to provide support, such as a doula, is associated with less use of analgesia [17], even though some studies have shown no differences in analgesia use [9, 18].

The CBD project in Gothenburg, Sweden, was established in 2008 by the community association, Födelsehuset/Mammaforum (‘Childbirth House/Mother Forum’) and has since provided CBDs during labour and birth for language assistance and support to migrant women not fluent in Swedish. Bilingual women from migrant communities trained in childbirth and labour support meet once or twice with women prior to the birth, provide a continuous presence and emotional and physical support as well as communication assistance during labour, and meet again with the women after the birth. Two qualitative evaluations, conducted in the early years of the program, indicated high levels of satisfaction among supported women [19] and midwives [20]. Improved communication and information-sharing and enhanced emotional and physical support were the most important outcomes reported. Since then around 1,400 women have received CBD support, however, the program has not yet been evaluated for its impact on birth outcomes. The current study fills an important gap in knowledge about the CBD model and complements an ongoing RCT of CBD support in Stockholm, Sweden – where the objective is to study women’s experiences of care and their emotional wellbeing [2] - by evaluating an already successfully implemented pragmatic model using a sufficient sample size for important labour and birth outcomes.

In Sweden, care during pregnancy, labour and birth is offered free of charge to all women. Less than one percent of all births take place outside hospitals. Registered midwives are the primary caregivers during normal labour and birth and where complications arise, an obstetrician will be consulted and/or assume responsibility for the care provided [21]. Care is regulated by national, regional and local guidelines and a midwife may care for one to four women in labour at the same time. Access to a language interpreter is mandated by Swedish law but the routines for language interpretation during labour and birth differ between hospitals and the use of tele-phone interpretation is most common. Interpreters are also most often provided on specific occasions when deemed necessary by staff, for instance to explain a procedure or change in the care strategy and not as a continuous means of communication support.

Migration of women of childbearing age (13–44 years) has increased most rapidly in Sweden from Somalia and from a number of Arabic-speaking countries (such as Iraq or Syria) and Eritrea [22]. Other migrant groups who have arrived in Sweden over the last decade include women from Central and Eastern Europe, as well as Central and South and East Asia [23]. Knowledge of the Swedish maternity care system, length of time in Sweden and fluency in Swedish vary between individuals and groups and communication difficulties when accessing care are also experienced to varying extents.

The aim of the current study was to compare birth outcomes for migrant women who received CBD support in labour, with birth outcomes for [1] migrant women who experienced usual care without CBD support, and [2] Swedish-born women giving birth during the same time period at the same hospitals. We hypothesised [1] that migrant women who received CBD support would use less analgesia and would have more non-instrumental vaginal births than migrant women who received usual intrapartum care without CBD support; and [2] that migrant women who received CBD support would not differ from Swedish-born women regarding analgesia use and mode of birth.

## Methods

### Design

This retrospective cohort study investigated data retrieved from a local register held by Mammaforum/ Födelsehuset in Gothenburg [24], and from two national registers: the Swedish Medical Birth register (MBR) [25] and Statistics Sweden [26] linked by each woman’s unique national identity number.The exposure of interest was CBD support and a range of obstetric outcomes of interest were investigated, as outlined below.

The MBR is based on mandatory notification of data from the medical records for all births in Sweden, and includes information on women’s obstetric background, maternal health before and during pregnancy, current pregnancy, labour and birth, and maternal and infant outcomes. The register was initiated in 1973, covers 99% of all births and the quality and validity of included data have been evaluated at three time points [27, 28]. Statistics Sweden produces and coordinates official statistics according to international standards for research [29] and provided information on migration and socioeconomic factors.

### Study setting and sample

Through Födelsehuset/Mammaforum (‘Childbirth House/Mother Forum’) in Gothenburg, women not fluent in Swedish were offered support by a CBD, bilingual in Swedish and the woman’s own language and well acquainted with the cultures of both countries. The ages of the CBDs ranged between 30 and 65 years and all had attended 8 days of certified training conducted by registered midwives with specific accreditation for CBD training. The course included basic anatomy, normal pregnancy and birth, relaxation techniques, pain relief, medical interventions, instrumental delivery, breast feeding, attachment and the newborn baby. Information about the CBD service was provided either when women participated in other activities organized by the community association or via a referral from the woman’s regular antenatal care midwife.

**Fig. 1:**
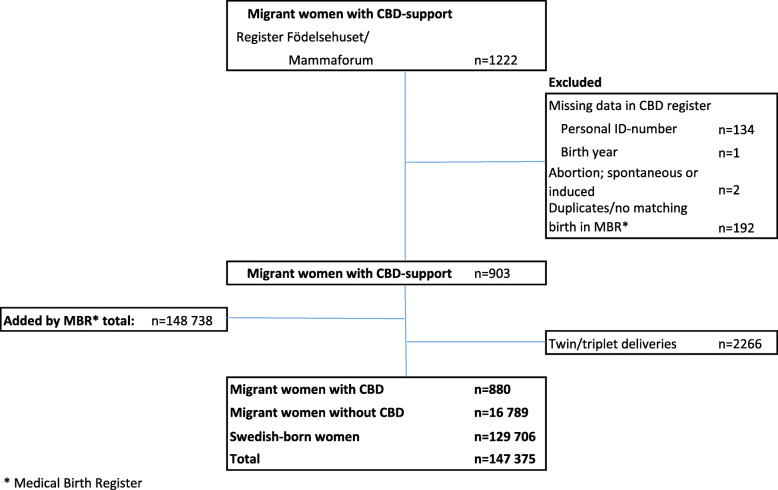
Selection of sample of births to migrant women with and without CBD support, and of Swedish born women.

The study sample included births to (1) migrant women who had received CBD during the period between 2008 and 2016, and for comparison, (2) migrant women from the same countries and who had given birth in the same hospitals during the study period but did not have support from a CBD during labour and birth and (3) all Swedish-born women who gave birth in the same hospitals during the same time-period as the migrant women. Births to migrant women with CBD support were identified through personal identification numbers available in a register kept by Födelsehuset/Mammaforum. After exclusion of births with incomplete personal ID, missing data on year and date of birth, and spontaneous or induced abortions, the file comprising migrant women with CBD support was sent to MFR and SCB for linkage to register data. Duplicates or no matching births in the MFR/SCB registers were excluded, the data were de-identified and matching births for the two comparison groups were identified and added to the data set (Fig. 1). For estimation of representativeness, data on maternal age, marital status, country of birth, BMI, smoking and parity for all births in Sweden during the time-period were also obtained.

### Exposure

CBD support for labour and birth was defined as being assigned to receive CBD support as reported in the register at Födelsehuset/Mammaforum. The CBD service included one to two preparatory meetings before the birth during which the doula and woman or couple could get acquainted with each other and the woman’s expectations and wishes for the coming birth could be shared, in addition to knowledge transfer about the birthing process and care during labour and birth in Sweden. During labour and birth the CBD provided continuous presence, emotional and physical and supported communication between the woman and the staff. The follow up visit after birth included reflections regarding the birthing experience, support in breast feeding and attachment, and information support as needed about Swedish social and other services.

### Outcomes

Outcome variables were retrieved from the MBR and included the following: induction of labour, use of epidural analgesia, nitrous oxide, bath, non-instrumental vaginal delivery, instrumental vaginal delivery (vacuum extraction *and* forceps), emergency caesarean, third or fourth degree perineal injury, length of mother’s hospital stay after the birth > 2 days and low Apgar score < 7 at five minutes.

### Other variables of interest

From Statistics Sweden we retrieved the following variables specifically for the year of the baby’s birth: maternal level of education (not completed compulsory school, compulsory school (9 years), upper secondary school (11–12 years), post-secondary education) and disposable income categorized into percentiles ≤ 25, 26–75, and ≥ 75 based on the household income per unit of consumption for all women giving birth in Sweden that year. Migration related variables retrieved from Statistics Sweden were maternal country of birth classified into region of birth, modified from Global Burden of Diseases (Sub-Saharan Africa, North Africa & Middle East, Central Europe, Eastern Europe & Central Asia, South & East Asia & Oceania, other) [30], reason for migration (work/education, refugee, family reunion, other) and length of residence in Sweden (< 2, 2–5 6–9, ≥ 10 years). From the MBR we retrieved the following variables: maternal age (< 25, 25–34, ≥ 35 years), marital status (married/cohabiting, single, other), previous cesarean section, previous stillbirth, year of the current birth, parity (nulliparous, parous), smoking in early pregnancy, chronic hypertension, diabetes (diabetes mellitus type one, two, or gestational diabetes), preeclampsia/eclampsia, pre-pregnancy body mass index (BMI) (underweight; <18.5 kg, normal weight (18.5–24.9 kg), overweight (25.0-29.9 kg,obesity; ≥30), number of antenatal visits (< 8, 8–12, > 12), gestational weeks (gwks) at birth (< 37, 37–41, ≥ 42) and birthweight (≤ 2500, 2501–4500 > 4500 grams).

### Statistical analyses

Univariate and multivariable logistic regression analyses were used to investigate possible associations between CBD support and birth outcomes and are reported as crude and adjusted odds ratios (ORs) with 95% confidence intervals (CIs). For transparency and to facilitate interpretation, adjustments were made in three steps. In the first model we adjusted for year of the current birth due to the long time span of the study (8 years), maternal age and marital status. In the second model we adjusted for the same variables and added hypertension, diabetes and BMI and in the third model, adjustments for disposable income and education were added. Missing values were considered not missing at random and analysed as a separate category so as not to lose cases in the final models. For the categorical variables used in the models; age, marital status, the first category (Table 1) was set as the reference. Analyses were conducted for nulliparous and multiparous women separately. Birth outcomes were also compared between migrant women with CBD support and Swedish-born women using the same strategy. In order to identify potential associations with CBD support in any particular groups of women, stratified analyses were performed in the following sub-groups: refugee background, Sub-Saharan or Middle East and North Africa (the largest groups) and length of residence in Sweden less than two years. All statistical analyses were performed using IBM Statistical Package for the Social Sciences 26.

**Table 1:** Background characteristics of the sample (*n* = 147,375)

	**Migrant women**					**Swedish-born women**		
	**CBD support** (*n* = 880)		**No CBD support** (*n* = 16,789)			(*n* = 129,706)		
	**n**	**%**	**n**	**%**	**p**^**a**^	**n**	**%**	**p**^**a**^
**Socioeconomic background**								
Age (years)					< 0.0001			< 0.0001
< 25	213	*24.2*	2829	*16.9*		15,757	*12.1*	
25–34	520	*59.1*	10,319	*61.5*		85,585	*66.0*	
≥ 35	147	*16.7*	3641	*21.7*		28,364	*21.9*	
Marital status					< 0.0001			< 0.0001
Married/cohabiting	621	*71.6*	14,251	*87.4*		115,804	*94.4*	
Single	91	*10.5*	751	*4.6*		2169	*1.8*	
Other	155	*17.9*	1302	*8*		4756	*3.9*	
Level of education					< 0.0001			< 0.0001
Not completed compulsory schooling^b^ (< 9 years)	255	*37.4*	2696	*17.6*		245	0.2	
Compulsory schooling (9 years)	101	*14.8*	1761	*11.5*		8317	6.4	
Upper secondary school (11–12 years)	143	*21.0*	4910	*32.1*		46,135	35.7	
Post-secondary education (vocational qualification & university)	183	*26.8*	5943	*38.8*		74,617	57.7	
Mother’s income					< 0.0001			< 0.0001
≤ 25 percentile	754	*89.5*	10,369	*63.2*		25,687	*19.8*	
25–75 percentile	82	*9.7*	4922	*29.4*		68,547	*52.9*	
≥ 75 percentile	6	*0.7*	1461	*8.7*		35,362	*27.3*	
**Migration**								
Region of birth					< 0.0001			
Sub-Saharan Africa	392	*44.6*	3595	*21.4*				
North Africa & Middle East	396	*45.1*	8348	*49.7*				
Central Europe Eastern Europe & Central Asia	76	*8.6*	3283	*19.6*				
South and East Asia & Oceania	10	*1.1*	1241	*7.4*				
Other	5	*0.6*	322	*1.9*				
Reason for immigration					< 0.0001			
Work/education	14	*1.7*	325	*2.1*				
Refugee	301	*36.4*	4817	*30.9*				
Family reunion	472	*57.1*	8215	*52.7*				
Other	39	*4.7*	2241	*14.4*				
Length of Residence					< 0.0001			
< 2 years	347	*41.2*	2966	*17.7*				
2–5 years	319	*37.9*	3741	*22.3*				
6–9 years	139	*16.5*	3186	*19.0*				
≥10 years	37	*4.4*	6885	*41.0*				
**Obstetric history**								
Previous caesarean section	98	*12.9*	1872	*11.7*	0.287	11,221	*8.7*	< 0.0001
Previous stillbirth	21	*2.4*	260	*1.5*	0.053	805	*0.6*	< 0.0001
**Present pregnancy**								
Parity					< 0.05			< 0.05
Nulliparous	379	*43.1*	6440	*38.4*		61,459	47.4	
Parous	500	56.9	10,349	*61.6*		68,247	*52.6*	
Smoking in early pregnancy	19	*2.2*	906	*5.6*	< 0.0001	7583	*6.2*	< 0.0001
Chronic hypertension	0	*0*	41	*0.2*	0.142	516	*0.4*	0.061
Diabetes Mellitus type 1 or 2	3	*0.3*	72	*0.4*	0.696	884	*0.7*	0.220
Gestational diabetes	12	*1.4*	414	*2.5*	< 0.05	1104	*0.9*	0.100
Pre-eclampsia/eclampsia	18	*2.0*	418	*2.5*	0.408	4059	*3.1*	0.065
Pre-pregnancy BMI (kg/m^2^)					< 0.0001			< 0.0001
Underweight (< 18.5)	24	*2.9*	425	*2.8*		2354	*2*	
Normal weight (18.5–24.9)	353	*42.6*	7904	*51.5*		73,085	*62.6*	
Overweight (25.0-29.9)	268	*32.3*	4676	*30.5*		27,953	*24*	
Obesity (≥ 30)	184	*22.2*	2334	*15.2*		13,317	*11.4*	
Antenatal care visits (No)					< 0.001			< 0.0001
< 8	115	*13.2*	2963	*18.1*		21,997	*17.0*	
8–12	544	*62.7*	10,132	*62*		82,289	*66.6*	
> 12	209	*24.1*	3258	*19.9*		19,334	*15.6*	
Gestational weeks (gwks)					< 0.0001			< 0.0001
Preterm < 37	19	*2.2*	942	*5.6*		7278	*5.6*	
Term 37–41	714	*81.1*	14,433	*86.0*		112,678	*86.9*	
Post-term ≥ 42	147	*16.7*	1410	*8.4*		9740	*7.5*	
Birth weight, (grams)					< 0.05			0.013
≤ 2500	26	*3.0*	888	*5.3*		4865	*3.8*	
2501–4500	834	*94.8*	15,566	*92.8*		119,528	*92.2*	
> 4500	20	*2.3*	319	*1.9*		5210	*4.0*	

^a^Differences between migrant women with CBD analysed

^b^According to the Swedish educational system

^c^Based on categorisation by Global Burden of Diseases

## Results

The final sample comprised 880 births to migrant women who had a CBD for labour and birth, 16,789 births to migrant women without a CBD present and 129,706 births to Swedish-born women; in total 147,375 births after the exclusion of those with incomplete data, spontaneous or induced abortion, births recorded twice, no matching birth in the MFR and twin or triplet births (Fig. 1).

Background characteristics of the study sample are shown in Table 1. Compared with both comparison groups, migrant women who had CBD support were: younger and more likely to be single, they had lower level of education and lower disposable income, they had higher rates of previous stillbirths, overweight and obesity, more were nulliparous, had more antenatal care visits than the recommended standard and fewer gave birth at term. Smoking was less common however, than among women in the comparison groups. Compared with migrant women without CBD support, those with CBD support were more likely to have sub-Saharan African origin, to be refugees or to having migrated for family reunion, and they had shorter duration of residence in Sweden. Chronic hypertension, diabetes type 1 and 2 and preeclampsia/eclampsia were similar in all three groups. The study sample was fairly similar to all births in Sweden during the same time period (the study sample excluded), but comprised a somewhat higher proportion of nulliparous women (45.6% vs. 43.1%, p < 0.0001), women of normal-weight (61.4% vs. 58.8%, p < 0.0001), and Swedish-born women (88.0% vs. 71.0%, p < 0.0001). The extent of missing background data varied between the respective groups and between variables, ranging from 0.1% to14%, with level of education varying even more (22.5%).

Associations between CBD support and labour, birth and infant outcomes in births to migrant women are shown in Table 2. Following adjustments, nulliparous migrant women with CBD support had decreased odds of having an epidural (aOR 0.64, CI 0.50–0.81) or of using a bath (aOR 0.64, CI 0.42–0.98) for labour pain compared with migrant women without such support. Parous migrant women supported by a CBD had increased odds of induction of labour (aOR 1.38, CI 1.08–1.76) and a hospital stay of more than 48 hours after the birth (aOR 1.19, CI 1.03–1.37) compared with migrant women without a CBD. No significant differences were found between the groups on the use of nitrous oxide, mode of birth, 3rd and 4th degree perineal injury, or low Apgar score at 5 minutes, either in primiparous or multiparous migrant women.

**Table 2:** Associations with labour and birth outcomes in nulliparous and parous migrant women with and without CBD support.

			**Migrant women with CBD**			**Migrant womenen without CBD**																
			***n* = 379**			***n* = 6440**																
			**n**	**%**		**n**		**%**	**OR**		**CI 95%**		**adjOR**^**a**^		**CI 95%**	**adjOR**^**b**^	**CI 95%**		**adjOR**^**c**^		**CI 95%**	
**Births to nulliparous women**																						
** Outcome**																						
Induction of labour			72	19.0		890		13.8	1.46		1.12–1.91		1.26		0.96–1.65	1.23	0.93–1.61		1.16		0.88–1.55	
Epidural analgesia			107	28.2		3760		39.6	0.60		0.48–0.76		0.61		0.48–0.77	0.61	0.48–0.77		0.64		0.50–0.81	
Nitrous oxide			281	74.1		5069		78.7	0.78		0.61–0.98		0.79		0.62-1.00	0.79	0.62–1.01		0.79		0.62–1.02	
Bath			25	6.6		757		11.8	0.53		0.35–0.80		0.56		0.37–0.85	0.57	0.38–0.87		0.64		0.42–0.98	
Non-instrumental vaginal birth^d^			267	70.4		4541		70.5	1.00		0.79–1.25		0.94		0.74–1.19	0.95	0.75–1.21		0.95		0.75–1.21	
Instrumental vaginal¹			34	9.0		596		9.3	1.04		0.73–1.46		1.12		0.79–1.58	1.11	0.79–1.59		0.99		0.70–1.43	
Emergency caesarean			67	17.7		972		15.1	1.21		0.92–1.59		1.20		0.91–1.58	1.17	0.88–1.55		1.21		0.91–1.61	
Perineal injury 3rd and 4th degree			6	1.6		155		2.4	0.65		0.29–1.48		0.73		0.32–1.68	0.74	0.32–1.69		0.59		0.25–1.39	
Apgar score < 7 at 5 minutes			9	2.4		112		1.8	1.37		0.69–2.72		1.28		0.64–2.58	1.26	0.62–2.53		1.26		0.62–2.57	
Hospital stay (mother) after birth > 2 days			206	54.9		3269		50.9	1.18		0.95–1.45		1.12		0.90–1.38	1.11	0.89–1.37		1.09		0.87–1.36	
			***n* = 500**			***n* = 10,349**			**Births to parous women**													
Induction of labour			89	17.8		1200		11.6	1.65		1.30–2.09		1.49		1.17–1.90	1.43	1.12–1.83		1.38		1.08–1.76	
Epidural analgesia			39	7.8		1212		11.7	0.64		0.46–0.89		0.63		0.45–0.88	0.64	0.46–0.89		0.80		0.57–1.13	
Nitrous oxide			321	64.2		7026		67.9	0.85		0.70–1.02		0.85		0.70–1.02	0.85	0.70–1.03		0.89		0.73–1.08	
Bath			16	3.2		339		3.3	0.98		0.59–1.63		1.02		0.61–1.71	1.08	0.65–1.82		1.25		0.74–2.11	
Non-instrumental vaginal birth^d^			428	85.6		8603		83.1	1.21		0.94–1.56		1.15		0.89–1.49	1.18	0.91–1.53		1.1		0.85–1.44	
Instrumental vaginal^d^			12	2.4		189		1.8	1.41		0.80–2.50		1.55		0.87–2.76	1.55	0.87–2.77		1.57		0.87–2.83	
Emergency caesarean			38	7.6		788		7.6	1.00		0.71–1.40		0.98		0.70–1.38	0.94	0.67–1.32		0.96		0.68–1.37	
Perineal injury 3rd and 4th degree			5	1.0		49		0.5	2.12		0.84–5.35		2.08		0.81–5.34	2.19	0.85–5.64		1.96		0.73–5.22	
Apgar score < 7 at 5 minutes			8	1.8		131		1.3	1.28		0.62–2.62		1.39		0.67–2.88	1.22	0.59–2.54		1.15		0.54–2.45	
Hospital stay (mother) after birth > 2 days			105	21.1		2096		20.3	1.06		0.93–1.22		1.03		0.89–1.18	1.01	0.88–1.17		1.19		1.03–1.37	

^a^ Adjusted for year of birth, maternal age, marital status ^b^ Adjusted for ^a^ and hypertension, diabetes and BMI ^c^ Adjusted for ^a^ and ^b^ and education and income

¹ Comparison group is instrumental vaginal *and* emergency caesarean births

Associations between CBD support and birth outcomes were also investigated in sub-groups of migrant women, nulliparous and parous women combined (data not shown). CBD support was associated with increased odds of induction of labour in women from Sub-Saharan Africa (CBD support 25.3% (*n* = 99); without CBD 18.7% (*n* = 673); aOR 1.38, CI 1.07–1.77), lower use of nitrous oxide in women from the Middle East and North Africa (CBD support 66.7% (*n* = 264); without CBD 71.6% (n = 5976); aOR 0.79, CI 0.63–0.99), lower use of epidural (CBD 19.6% (*n* = 68); without CBD 29.1% (*n* = 862); aOR 0.69, CI 0.52–0.92) and nitrous oxide (CBD support 64.8% (*n* = 225); without CBD 73.4% (n = 2177); aOR 0.78, CI 0.61–0.99) in women with short residence in Sweden (< 2 years).

Birth outcomes for migrant women who received CBD support were also compared with those of the Swedish-born women (Table 3). Both nulliparous and parous migrant women with CBD support had lower odds for having an epidural (aOR 0.50, CI 0.39–0.64 and aOR 0.40, CI 0.28–0.58) or using nitrous oxide (aOR 0.71, CI 0.54–0.92 and aOR 0.68, CI 0.54–0.84), Nulliparous women with CBD support also had lower odds of using a bath for pain relief (aOR 0.55, CI 0.36–0.85), higher odds of an emergency caesarean (aOR 1.43, CI 1.05–1.94) and of a maternal hospital stay > 48 hours (aOR 1.31, CI 1.04–1.64) than the Swedish-born women. The odds for 3rd and 4th degree perineal injury and low Apgar score were similar between the groups.

**Table 3:** Associations with labour and birth outcomes in nulliparous and parous women comparing migrant women with CBD support and Swedish born women

	**Migrant women with CBD**				**Swedish born women**																		
	**(*n* = 379)**				**(*n*= 61,459)**																		
	**n**	**%**			**n**		**%**		**OR**	**CI 95%**		**adjOR**^**a**^		**CI 95%**		**adjOR**^**b**^		**CI 95%**		**adjOR**^**c**^		**CI 95%**	
**Births to nulliparous women**																							
** Outcome**																							
Induction of labour	72	19.0			8368		13.6		1.49	1.15–1.93		1.43		1.10–1.86		1.43		1.10–1.86		1.18		0.87–1.58	
Epidural analgesia	107	28.2			26,499		43.1		0.52	0.42–0.65		0.47		0.38–0.59		0.47		0.37–0.59		0.50		0.39–0.64	
Nitrous oxide	281	74.1			50,972		82.9		0.59	0.47–0.74		0.61		0.48–0.77		0.61		0.48–0.77		0.71		0.54–0.92	
Bath	25	6.6			8907		14.5		0.42	0.28–0.63		0.44		0.29–0.66		0.44		0.29–0.66		0.55		0.36–0.85	
Non-instrumental birth	267	70.4			44,108		71.8		0.94	0.75–1.17		0.82		0.65–1.02		0.82		0.65–1.03		0.89		0.69–1.14	
Instrumental vaginal¹	34	9.0			6521		10.6		0.90	0.65–1.26		1.05		0.75–1.47		1.05		0.75–1.47		0.99		0.68–1.43	
Emergency caesarean	67	17.7			7841		12.8		1.47	1.13–1.91		1.62		1.24–2.12		1.61		1.22–2.11		1.43		1.05–1.94	
Perineal injury 3rd and 4th degree	6	1.6			1546		2.5		0.62	0.28–1.40		0.84		0.37–1.88		0.84		0.37–1.88		0.91		0.39–2.16	
Apgarscore < 7 at 5 minutes	9	2.4			1047		1.7		1.40	0.72–2.72		1.40		0.71–2.73		1.41		0.72–2.75		1.28		0.60–2.72	
Hospital stay (mother) after birth > 2 days	206	54.9			30,541		49.8		1.23	1.00-1.51		1.36		1.10–1.67		1.35		1.10–1.67		1.31		1.04–1.64	
	**(*n* = 500)**				**(*n* = 68,247)**				**Births to parous women**														
Induction of labour	89	17.8			7769		11.4		1.69	1.34–2.12		1.45		1.15–1.83		1.28		1.01–1.62		1.04		0.78–1.38	
Epidural analgesia	39	7.8			9940		14.6		0.50	0.36–0.69		0.46		0.33–0.64		0.42		0.30–0.59		0.40		0.28–0.58	
Nitrous oxide	321	64.2			50,413		73.9		0.63	0.53–0.76		0.65		0.54–0.78		0.66		0.55–0.79		0.68		0.54–0.84	
Bath	16	3.2			3790		5.6		0.56	0.34–0.93		0.65		0.39–1.07		0.66		0.40–1.09		0.88		0.50–1.53	
Non-instrumental births^d^	428	85.6			57,502		84.3		1.11	0.87–1.43		1.08		0.84–1.40		1.22		0.95–1.58		1.17		0.86–1.58	
Instrumental vaginal¹	11	2.4			1435		2.1		1.21	0.70–2.10		1.30		0.74–2.27		1.28		0.73–2.24		1.38		0.70–2.72	
Emergency caesarean	38	7.6			4147		6.1		1.27	0.91–1.77		1.27		0.91–1.78		1.07		0.76–1.50		0.97		0.64–1.46	
Perineal injury 3rd and 4th degree	5	1.0			578		0.8		1.18	0.49–2.87		1.48		0.61–3.61		1.53		0.63–3.75		2.77		0.95–8.04	
Apgar score < 7 at 5 minutes	8	1.8			694		1.1		1.60	0.79–3.22		1.53		0.75–3.11		1.27		0.62–2.60		1.05		0.45–2.47	
Hospital stay after birth (mother) > 2 days	105	21.1			14,056		20.6		1.03	0.83–1.28		1.08		0.87–1.35		1.00		0.80–1.25		0.95		0-73-1.23	

^a^ Adjusted for year of birth, maternal age, marital status ^b^ Adjusted for ^a^ and hypertension, diabetes and BMI ^c^ Adjusted for ^a^ and ^b^ and education and income

^d^Comparison group is instrumental vaginal *and* emergency caesarean birth

## Discussion

Migrant nulliparous women and those with short length of residence who received labour and birth support from a CBD used less epidural analgesia and were less likely to use a bath for pain relief, compared with migrant women without such support. The support from a CBD did not however increase migrant women’s probability of a non-instrumental vaginal birth. Compared with Swedish-born women, nulliparous migrant women supported by a CBD, were more likely to have an emergency caesarean, longer hospital stays and to have lower odds of pain relief during labour.

### Strengths and limitations

This is the first register study to investigate the associations between CBD support for migrant women and a number of labour and birth outcomes in a multicultural setting. Linking data over a nine year period of CBD implementation with high quality national registers made it possible to include a sufficient number of CBD-supported women to compare their outcomes with the outcomes both for migrant women without such support and with Swedish-born women giving birth in the same hospitals during the same time period. Limited numbers in some sub-group analyses meant that instrumental vaginal births could not be analysed for refugee women separately, which is a limitation. A register study design enabled a focus on routinely collected obstetric outcomes. Although the wellbeing of women and their experiences of labour care and support are important, this study was unable to investigate these outcomes. The aforementioned ongoing RCT was designed to address these experiential outcomes.

We cannot dismiss the possibility of residual confounding although we strived to adjust for relevant confounding factors where possible. In addition, bearing in mind the differences in parity, BMI and language groups between the study sample and all births in Sweden during the same time period, the study findings cannot be generalized to the whole population or to immigrants in Sweden as a whole.

### Interpretation

Notably, the results must be interpreted bearing in mind the selection of women for CBD support in labour and birth evident in this study. Our findings confirm that the CBD services had reached the women intended, in terms of their more vulnerable socioeconomic circumstances and migration related factors such as recent arrival, and/or their increased risks of adverse birth outcomes and experiences.

The finding of an overall lower use of analgesia among the migrant women supported by a CBD, compared to migrant women without CBD services, confirms one of our study hypotheses. The finding could be viewed from two perspectives. Interpreted in light of systematic reviews by Hodnett et al. [31] and Bohren et al. [17] showing that continuous support for women during childbirth contributes to lower analgesia use, our findings may indicate a positive impact of the presence of a CBD and that women actually did not need an epidural. With a CBD present for continuous support, providing calmness and safety and improved communication during labour and birth, the prerequisites for women to manage labour pain and freely choose pain relief have most likely improved, and choices to abstain from pain relief may be more well-grounded. In comparison with Swedish-born women, the migrant women with CBD support also used less pain relief. This may, in the same way, be associated with the continuous presence of the CBD, indicating that even Swedish-born women would benefit from enhanced continuous support during labour and birth. On the other hand, our findings might be interpreted as evidence that migrant women miss out on available pain relief [32, 33]. In line with the latter interpretation, two recent Norwegian register-based studies found lower use of epidural analgesia among women with similar characteristics as many of those receiving CBD support in our study, namely refugees, newly arrived women and women of Sub-Saharan origin [34, 35]. Qualitative studies conducted with migrant women themselves may be able to achieve more in-depth and nuanced perspectives on the role of CBD support for the adequacy of pain relief.

The hypothesis that CBD support would increase non-instrumental vaginal births in migrant women was not confirmed. Previous cohort studies conducted in the United States and the United Kingdom have indicated reduced likelihood of operative birth when doula support has been provided [8, 12, 14, 36, 37], however not consistently [9, 18, 38]. The reason our results did not demonstrate an increase in non-instrumental births may partly be due to differences in policies and “culture” regarding caesarean sections and non-instrumental vaginal births. With an overall caesarean section rate of 17%, Sweden has a substantially lower rate [39] compared with the countries where previous studies were conducted; the United Kingdom 25–30% [39], the United States 33% [40], leaving less room to reduce caesarean sections further in the Swedish context by means of CBD support to migrant women. CBD support was associated in fact with *increased* odds of emergency caesarean section in nulliparous migrant women when compared with Swedish-born women, though not in parous women or when compared with migrant women without CBD support. This finding in nulliparous women might be interpreted as due to the increased risk of adverse pregnancy outcomes and poorer health particularly among women with refugee background [41] in spite of the adjustments made for some risks, *or* alternatively to improved communication and responses to women’s need for, or wishes regarding intervention [19, 20].

Given that the migrant women who received CBD support constituted a socioeconomically and obstetrically vulnerable group (more single status, lower levels of education and income, refugee background and high BMI) [42], one might have expected higher odds of adverse birth outcomes in general within this group than in those without CBD support or in the Swedish-born women. The presence of a CBD strengthening communication between the woman and health professionals during labour as well as providing relaxation support, could be explanatory, as both these factors are linked to higher prevalence of non-instrumental vaginal birth [7, 43]. In light of this, offering CBD services to selected migrant women could be seen as an intervention addressing both inequalities in health and inequities in care, problems often highlighted in recent years [42, 44]. Our finding of more inductions of labour among migrant women with CBD support, and in particular among Somali-born women might suggest a similar mechanism. With the maternal characteristics reported in this study, and previously found adverse pregnancy outcomes within similar groups [33, 45], strengthened communication via a CBD may have better enabled adequate surveillance and timely decisions for intervention.

Comparing the birth outcomes in the current study with similar studies in different countries highlights the complexities of cross-country comparisons in the context of different health care structures and accessibility, as well as differing health outcomes. So far, most previous studies of CBD support have been conducted in the United States and a few in the United Kingdom. Additional RCTs investigating both women’s experiences and obstetric outcomes are needed. To our knowledge, only one RCT is currently being conducted in a setting similar to the one presented here [2], however, the results of other ongoing intervention studies, albeit with other study designs and outcome measures in Norway [46], and Australia [47], will make valuable additions to the evidence base.

## Conclusions

CBD support appears to have the potential to reduce analgesia use in migrant women with vulnerability to adverse outcomes, as in this study. It may also be that some necessary interventions were facilitated by the enhanced communication made possible by the presence of a CBD. Further studies of the effect of CBD support on mode of birth and other obstetric outcomes as well as on migrant women’s experiences and well-being are needed.

## Data Availability

The data sets generated and analysed during the current study are not publicly available due to confidentiality regulations, but may be available from the corresponding author on reasonable request.

## Abbreviations

CBD: Community-based Bilingual Doula
MBR: The Swedish Medical Birth Register
SCB: Statistics Sweden
aOR: Adjusted odds ratio
CI: Confidence intervals
